# Microarray analysis identifies a set of CXCR3 and CCR2 ligand chemokines as early IFNβ-responsive genes in peripheral blood lymphocytes *in vitro*: an implication for IFNβ-related adverse effects in multiple sclerosis

**DOI:** 10.1186/1471-2377-6-18

**Published:** 2006-05-19

**Authors:** Jun-ichi Satoh, Yusuke Nanri, Hiroko Tabunoki, Takashi Yamamura

**Affiliations:** 1 Department of Bioinformatics, Meiji Pharmaceutical University, 2-522-1 Noshio, Kiyose, Tokyo 204-8588, Japan; 2 Department of Immunology, National Institute of Neuroscience, NCNP, 4-1-1 Ogawahigashi, Kodaira, Tokyo 187-8502, Japan

## Abstract

**Background:**

A substantial proportion of multiple sclerosis (MS) patients discontinue interferon-beta (IFNβ) treatment due to various adverse effects, most of which emerge at the early phase after initiation of the treatment and then diminish with time. At present, the molecular mechanism underlying IFNβ-related adverse effects remains largely unknown. The aim of this study is to identify a comprehensive list of early IFNβ-responsive genes (IRGs) in peripheral blood mononuclear cells (PBMC) that may play a key role in induction of adverse effects.

**Methods:**

Total RNA of PBMC exposed to 50 ng/ml recombinant human IFNβ for 3 to 24 hours *in vitro *was processed for cDNA microarray analysis, followed by quantitative real-time RT-PCR analysis.

**Results:**

Among 1,258 genes on the array, IFNβ elevated the expression of 107 and 87 genes, while it reduced the expression of 22 and 23 genes at 3 and 24 hours, respectively. Upregulated IRGs were categorized into conventional IFN-response markers, components of IFN-signaling pathways, chemokines, cytokines, growth factors, and their receptors, regulators of apoptosis, DNA damage, and cell cycle, heat shock proteins, and costimulatory and adhesion molecules. IFNβ markedly upregulated CXCR3 ligand chemokines (SCYB11, SCYB10 and SCYB9) chiefly active on effector T helper type 1 (Th1) T cells, and CCR2 ligand chemokines (SCYA8 and SCYA2) effective on monocytes, whereas it downregulated CXCR2 ligand chemokines (SCYB2, SCYB1 and IL8) primarily active on neutrophils.

**Conclusion:**

IFNβ immediately induces a burst of gene expression of proinflammatory chemokines *in vitro *that have potential relevance to IFNβ-related early adverse effects in MS patients *in vivo*.

## Background

Multiple sclerosis (MS) is an inflammatory demyelinating disease of the central nervous system (CNS) white matter mediated by an autoimmune process, whose development is triggered by a complex interplay of both genetic and environmental factors [1]. Administration of interferon-gamma (IFNγ) induced acute relapses, along with activation of the systemic immune response [2], suggesting that T-lymphocytes producing proinflammatory T helper type 1 (Th1) cytokines play a pivotal role in the immunopathogenesis of MS. In contrast, interferon-beta (IFNβ) significantly reduces the frequency of clinical exacerbations and delays the progression of disability in relapsing-remitting MS (RRMS), accompanied by a reduction in the number of new brain lesions on MRI [3,4]. Furthermore, an early initiation of IFNβ delays the conversion to clinically definite MS in the patients who experienced a first demyelinating event [5]. Although the precise mechanism underlying therapeutic effects of IFNβ on MS remains to be fully elucidated, previous studies proposed several possibilities, including the inhibition of Th1 cell development [6], induction of Th2 immune deviation [7], restoration of function of the disrupted blood-brain barrier [8], and downregulation of IFNγ-induced expression of class II major histocompatibility complex (MHC) molecules [9].

Although clinical benefits of IFNβ in MS are meaningful, approximately one-third of the patients receiving IFNβ therapy suffered from a higher or identical annual relapse rate on treatment [10]. New lesion formation on MRI during the treatment correlates with poor response to IFNβ [11]. Furthermore, a substantial proportion of the patients discontinued IFNβ treatment because of various adverse effects, including skin reactions, flu-like symptoms, leukocytopenia, liver dysfunction, depression and amenorrhea [12]. The molecular mechanisms accounting for IFNβ-related adverse effects remain unknown, although most of these emerge at the early phase after initiation of the treatment, and then diminish with time [12]. At present, no biologically relevant markers capable of predicting either therapeutic or detrimental responses of IFNβ in MS are available [13].

DNA microarray technology is a novel approach that allows us to systematically monitor the expression of a large number of genes. It has given new insights into the complexity of molecular interactions promoting the autoimmune process in MS [14]. IFNβ induces a complex pattern of gene regulation in peripheral blood mononuclear cells (PBMC) of MS [15]. Recently, we studied the gene expression profile of CD3^+ ^T cells isolated from PBMC of 13 Japanese MS patients before and after treatment with IFNβ-1b by analyzing a custom cDNA microarray containing a set of well-annotated, immunologically relevant genes. IFNβ upregulates the expression of 7 IFNβ-responsive genes (IRGs) during the treatment [16]. A following study performed on RRMS patients receiving IFNβ-1a supported our observations [17]. More recently, we found that the vast majority of genes expressed in CD3^+ ^T cells differentially between 72 untreated MS patients and 22 healthy controls are categorized into apoptosis regulators [18]. Regarding the gene expression profile of IFNβ responders in MS, baseline levels of IL-12p35 mRNA are significantly lower in the responders [19]. Downregulation of IL-8 expression in PBMC during IFNβ treatment distinguishes the responders from nonresponders in RRMS [20]. IFNβ responders differ from nonresponders in the kinetics of expression of IRGs at 3 and 6 months after starting the treatment [21]. A three-dimensional model of gene triplets detected by RT-PCR analysis predicts IFNβ response status in RRMS [22]. However, all of previous observations do not clearly illustrate the molecular basis of complex biological effects of IFNβ in MS. Furthermore, no databases of immediate early IRGs in PBMC are currently available.

The present study is designed to identify a comprehensive list of immediate early IRGs in PBMC with potential relevance to IFNβ-related early adverse effects in MS.

## Methods

### Treatment of peripheral blood lymphocytes with IFNβ

PBMC were isolated from heparinized blood by centrifugation on a Ficoll density gradient. PBMC were suspended at 5 × 10^6 ^cells/ml in RPMI 1640 medium containing 10% fetal bovine serum, 2 mM L-glutamine, 55 μM 2-mercaptoethanol, 100 U/ml penicillin, and 100 μg/ml streptomycin (culture medium). The cells were incubated in a 5%CO_2_/95% air incubator at 37°C for 3 hours to characterize the immediate response or for 24 hours to detect the early response, in the culture medium with or without inclusion of 50 ng/ml recombinant human IFNβ (a specific activity of = 2 × 10^7 ^units/mg, PeproTech, London, UK), 50 ng/ml recombinant human IFNγ (a specific activity of = 2 × 10^7 ^units/mg, PeproTech), 50 ng/ml recombinant human TNFα (a specific activity of = 2 × 10^7 ^units/mg, PeproTech), or 50 ng/ml recombinant human IL-1β (a specific activity of = 1 × 10^7 ^units/mg, PeproTech). They were then processed for RNA preparation as described previously [16,18,21]. Written informed consents were obtained from the subjects involved in the present study according to the form approved by the Ethics Committee of National Center of Neurology and Psychiatry (NCNP), Tokyo, Japan. The samples of the subjects #1, #2 and #4 were processed for both microarray and real-time RT-PCR analysis, while those of the subject #3 were studied only by real-time RT-PCR analysis.

### Quantitative real-time RT-PCR analysis

DNase-treated total RNA was processed for cDNA synthesis using oligo(dT)_12–18 _primers and SuperScript II reverse transcriptase (Invitrogen, Carlsbad, CA). cDNA was amplified by PCR in LightCycler ST300 (Roche Diagnostics, Tokyo, Japan) using SYBR Green I dye and the primer sets listed in Table 1. To calibrate the concentration of mRNA levels in test cDNA samples, serially-diluted purified PCR products generated by conventional RT-PCR (a 10-fold dilution from 1 pg/ml to 0.0001 pg/ml) were amplified in parallel. The levels of expression of target genes were standardized against those of the glyceraldehyde-3-phosphate dehydrogenase (G3PDH) gene detected in the identical cDNA samples. The assays were performed in triplicate measurements of the same sample and the results were expressed as the average with standard error.

### cDNA microarray analysis

The present study utilized a custom microarray containing duplicate spots of 1,258 cDNA immobilized on a poly-L-lysine-coated slide glass (Hitachi Life Science, Kawagoe, Saitama, Japan) [16,18,21]. They were prepared by PCR of well-annotated genes, selected from cytokines, growth factors and their receptors, apoptosis regulators, oncogenes, transcription factors, cell cycle regulators and housekeeping genes. The complete gene list is shown in Additional file 1. Five μg of purified RNA was *in vitro *amplified and antisense RNA (aRNA) was processed for microarray analysis. aRNA derived from IFNβ-treated PBMC was labeled with a fluorescent dye Cy5, while aRNA of untreated PBMC was labeled with Cy3 by reverse transcriptase reaction. The arrays were hybridized at 62°C for 17 hours in the hybridization buffer containing equal amounts of Cy3- or Cy5-labeled cDNA, and they were then scanned at two different photomultiplier tube (PMT) gains by the ScanArray 5000 scanner (GSI Lumonics, Boston, MA). The data were analyzed by using the QuantArray software (GSI Lumonics). The fluorescence intensities (FI) of individual spots were quantified following global normalization between Cy3 and Cy5 signals. The average of FI of duplicate spots was calculated, then the ratio of FI of Cy5 signal versus FI of Cy3 signal exceeding 2.0 was defined as significant upregulation, whereas the ratio smaller than 0.5 was considered as substantial downregulation. The impact of inter-experiment variability was validated by analyzing a scatter plot (see Additional file 2).

The IFN-regulated expression of the genes we identified was verified by computerized search of PubMed database and IFN Stimulated Gene (ISG) database [23].

## Results

### Microarray analysis identified immediate early IFNβ-responsive genes in PBMC

Among 1,258 genes on the array, IFNβ treatment for 3 hours elevated the expression of 107 genes in PBMC isolated from a 46 year-old healthy man (the subject #1), while it reduced the expression of 22 genes (see Additional files 3 and 4 for the complete list). IFNβ treatment for 24 hours upregulated 87 genes and downregulated 23 genes (see Additional files 5 and 6 for the complete list). Sixty-nine genes were upregulated at both 3 and 24 hours, while only two genes such as FOS and IL1A were downregulated at both. The IRGs upregulated at both time points contained 11 *in vivo *IRGs reported previously by us [16], including IFIT1 (IFI56), ISG15 (G1P2), IFIT4 (IFI60), IFI27, G1P3 (IFI6-16), IRF7, ABCB2 (TAP1), ATF3, IFITM1 (IFI17), SULT1C1, and TNFAIP6, whose expression was elevated in T cells and non-T cells *ex vivo*, isolated from 13 RRMS patients during IFNβ treatment for 3 to 6 months. Top 20 most significant genes, either upregulated or downregulated, are listed in Table 2 and Table 3, respectively. All of top 20 upregulated genes were found as known IRGs identified in various cell types by searching through PubMed and ISG databases.

The upregulated IRGs in the complete lists (see Additional files 3 and 5) were classified into several functional categories following; (i) conventional IFN-response markers (n = 12), (ii) components of classical and Toll-like receptor (TLR)-dependent IFN-signaling pathways (n = 12), (iii) chemokines and their receptors (n = 11), (iv) cytokines, growth factors and their receptors (n = 17), (v) apoptosis, DNA damage, and cell cycle regulators (n = 29), (vi) heat shock proteins (n = 9), and (vii) costimulatory and adhesion molecules (n = 7) (Table 4). The chemokine and chemokine receptor group included both CXC and CC chemokines and their receptors, such as SCYB11 (CXCL11, I-TAC), SCYB10 (CXCL10, IP-10), SCYA8 (CCL8, MCP2), SCYB9 (CXCL9, MIG), SCYA2 (CCL2, MCP1), CCR5, SCYA4 (CCL4, MIP1B), IL8RB (CXCR2), SCYA3 (CCL3, MIP1A), SCYA19 (CCL19, MIP3B) and SCYA13 (CCL13, MCP4). It is worthy to note that both CXCR3 ligand chemokines (SCYB11, SCYB10 and SCYB9) and CCR2 ligand chemokines (SCYA10 and SCYA2) were clustered in top 20 genes greatly elevated at 3 and 24 hours of IFNβ treatment (Table 2). With respect to top 20 downregulated genes, four genes such as SCYB2 (CXCL2, GRO2), SCYB1 (CXCL1, GRO1), IL8 (SCYB8, CXCL8), and SCYA24 (eotaxin-2) were categorized into the chemokine group (Table 3). Among them, SCYB2, SCYB1 and IL8, whose expression was reduced immediately at 3 hours of IFNβ treatment, belong to CXCR2 ligand chemokines. Although the analysis in the present study was a single microarray for each sample design, the results from two additional subjects, including a 28 year-old healthy man (the subject #2) and a 27 year-old woman with RRMS who was a dropout of IFNβ treatment due to induction of frequent severe relapses (the subject #4), verified the observations of immediate early induction of CXCR3 ligand and CCR2 ligand chemokine genes in PBMC by exposure to IFNβ, supporting the reproducibility of these results (see Additional file 7).

### Real-time RT-PCR analysis validated IFNβ-regulated expression of IRGs identified by microarray analysis

**Figure 1:**
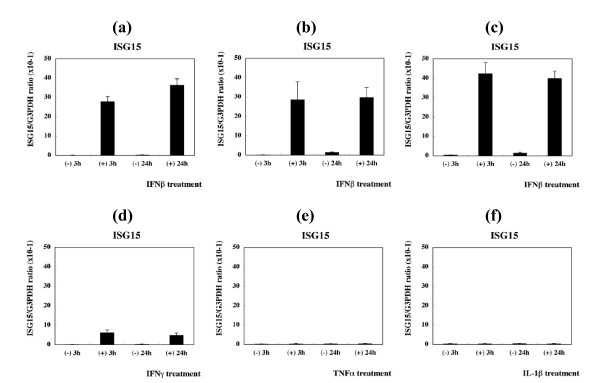
*Real-time RT-PCR analysis of ISG15 expression in PBMC*. PBMC derived from three distinct healthy subjects numbered #1 (a 46 year-old man), #2 (a 28 year-old man), and #3 (a 42 year-old woman) were incubated for 3 hours or 24 hours in the culture medium with (+) or without (-) inclusion of recombinant human IFNβ, IFNγ, TNFα or IL-1β at a concentration of 50 ng/ml each. cDNA was processed for real-time PCR analysis using specific primers listed in Table 1. The levels of expression of ISG15 are standardized against those of the glyceraldehyde-3-phosphate dehydrogenase (G3PDH) gene detected in identical cDNA samples. The assays were performed in triplicate measurements of the same sample, and the results were expressed as the average with standard error. The panels represent the expression of ISG15 in (a) #1, IFNβ; (b) #2, IFNβ; (c) #3, IFNβ; (d) #1, IFNγ; (e) #1, TNFα; and (f) #1, IL-1β.

**Figure 2:**
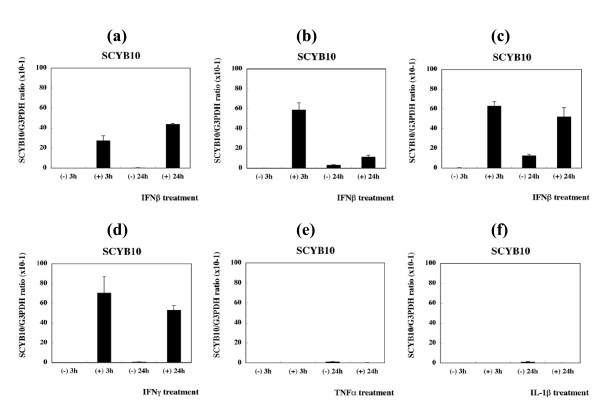
*Real-time RT-PCR analysis of SCYB10 expression in PBMC*. See the footnote of Figure 1. The panels represent the expression of SCYB10 in (a) #1, IFNβ; (b) #2, IFNβ; (c) #3, IFNβ; (d) #1, IFNγ; (e) #1, TNFα; and (f) #1, IL-1β.

**Figure 3:**
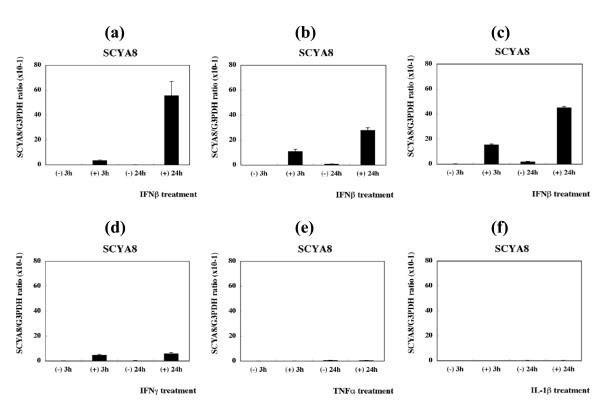
*Real-time RT-PCR analysis of SCYA8 expression in PBMC*. See the footnote of Figure 1. The panels represent the expression of SCYA8 in (a) #1, IFNβ; (b) #2, IFNβ; (c) #3, IFNβ; (d) #1, IFNγ; (e) #1, TNFα; and (f) #1, IL-1β.

**Figure 4:**
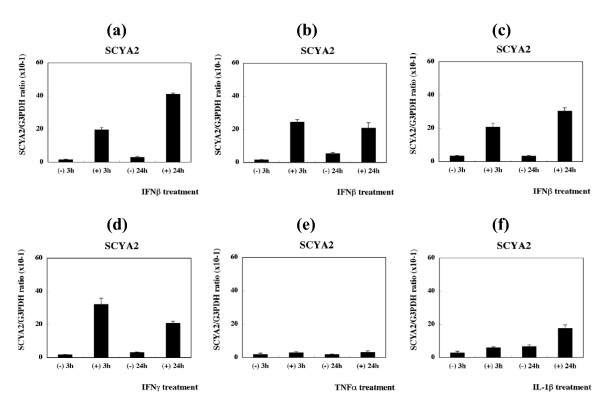
*Real-time RT-PCR analysis of SCYA2 expression in PBMC*. See the footnote of Figure 1. The panels represent the expression of SCYA2 in (a) #1, IFNβ; (b) #2, IFNβ; (c) #3, IFNβ; (d) #1, IFNγ; (e) #1, TNFα; and (f) #1, IL-1β.

**Figure 5:**
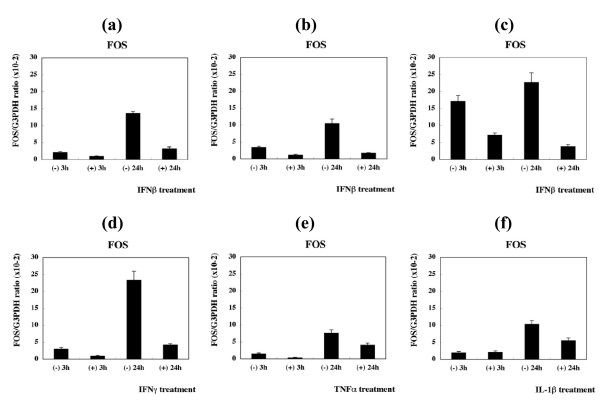
*Real-time RT-PCR analysis of FOS expression in PBMC*. See the footnote of Figure 1. The panels represent the expression of FOS in (a) #1, IFNβ; (b) #2, IFNβ; (c) #3, IFNβ; (d) #1, IFNγ; (e) #1, TNFα; and (f) #1, IL-1β.

**Figure 6:**
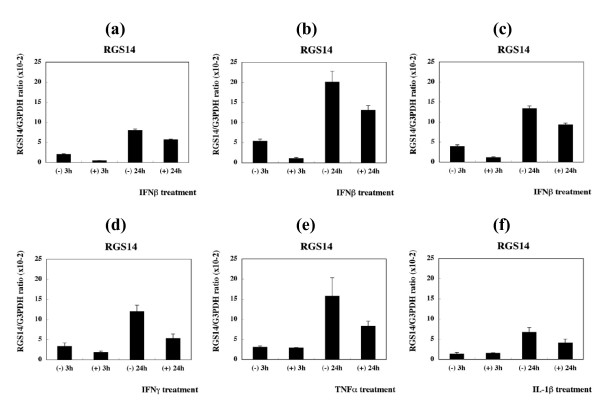
*Real-time RT-PCR analysis of RGS14 expression in PBMC*. See the footnote of Figure 1. The panels represent the expression of RGS14 in (a) #1, IFNβ; (b) #2, IFNβ; (c) #3, IFNβ; (d) #1, IFNγ; (e) #1, TNFα; and (f) #1, IL-1β.

**Figure 7:**
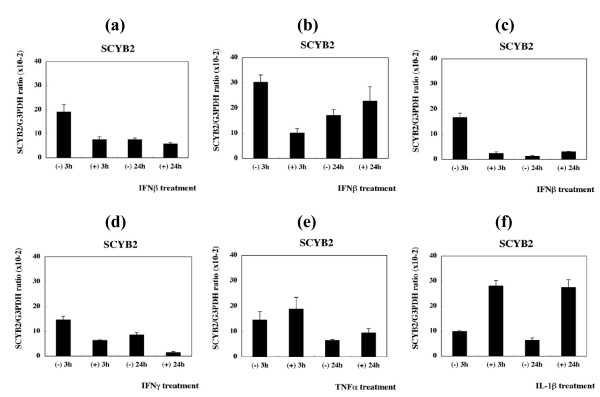
*Real-time RT-PCR analysis of SCYB2 expression in PBMC*. See the footnote of Figure 1. The panels represent the expression of SCYB2 in (a) #1, IFNβ; (b) #2, IFNβ; (c) #3, IFNβ; (d) #1, IFNγ; (e) #1, TNFα; and (f) #1, IL-1β.

Although the microarray we utilized contains total 64 spots of the G3PDH gene (see Additional file 1), G3PDH was neither identified as a significantly upregulated nor a downregulated gene in the microarray analysis, suggesting that G3PDH represents a reliable housekeeping gene in gene expression analysis of PBMC following treatment with IFNβ. Therefore, quantitative real-time RT-PCR analysis was performed by evaluating the levels of expression of target genes standardized against those of G3PDH detected in the identical cDNA samples. It verified the key observations of microarray analysis, such as marked upregulation of ISG15, the prototype of IRGs (Figure 1a–c), and great elevation of SCYB10, SCYA8 and SCYA2 (Figures 2, 3, 4a–c) in PBMC at both 3 and 24 hours of IFNβ treatment. Furthermore, the quantitative analysis confirmed substantial downregulation of FOS at both time points (Figure 5a–c), and RGS14 and SCYB2 predominantly at 3 hours (Figures 6, 7a–c). Exposure of PBMC to IFNγ greatly elevated the expression of SCYB10 and SCYA2, and to a lessor extent, ISG15 and SCYA8 at both time points (Figures 1, 2, 3, 4d), suggesting a functional overlap in induction of CXCR3 ligand and CCR2 ligand chemokines between type I and type II IFN signaling pathways. In contrast, TNFα and IL-1β the prototype of proinflammatory cytokines, did not at all elevate the levels of expression of ISG15, SCYB10 or SCYA8 (Figures 1, 2, 3e, f), while IL-1β significantly (*p *= 0.041 at 3 hours and *p *= 0.004 at 24 hours by two-sided paired t-test) but TNFα only marginally (*p *= 0.2102 at 3 hours and *p *= 0.0825 at 24 hours by two-sided paired t-test) upregulated SCYA2 expression (Figure 4e, f). Treatment with IFNγ, TNFα or IL-1β reduced the levels of FOS and RGS14 substantially at 24 hours (Figures 5, 6d–f). IFNγ reduced the expression of SCYB2, whereas TNFα and IL-1β elevated its levels at both time points, suggesting differential regulation of SCYB2 gene expression in PBMC by exposure to distinct cytokines (Figure 7d–f). The IFNβ-regulated gene expression pattern was similar among PBMC derived from three distinct healthy subjects #1, #2 and #3, supporting the reproducibility of these observations (Figures 1, 2, 3, 4, 5, 6, 7a–c).

## Discussion

IFNs are a family of cytokines that mediates antiviral, antiproliferative and immunoregulatory activities. Type I IFNs, IFNα and β, are produced principally by virus-infected host cells, whereas type II IFN, IFNγ, is produced by activated T cells and natural killer (NK) cells. Type I IFNs activate JAK protein tyrosine kinases associated with the cell surface receptors for IFNs, leading to formation of the complex of signal transducer and activator of transcription (STAT) molecules with the IFN regulatory factor (IRF) family of transcription factors. The STAT/IRF complex translocates into the nucleus, and binds to the DNA sequences termed the IFN-stimulated response element (ISRE) or the IRF-recognition element (IRE). This binding subsequently activates transcription of a wide variety of IFN-responsive genes (IRGs) as well as the genes of type I and type II IFNs, leading to the biological responses triggered by the IFNs [24]. Both type I and type II IFNs enhance the expression of class I and class II MHC molecules [25]. Among nine distinct IRFs, IRF7 and IRF3 play a central role in induction of type I IFN genes via the virus-activated MYD88-independent pathway or Toll-like receptor (TLR)7, 8 or 9-activated MYD88-dependent pathway [26], while IRF1 plays more active roles in induction of IFNγ-target genes essential for Th1-type immune response [25].

The present study by analyzing DNA microarray characterized a comprehensive list of immediate early IRGs in PBMC *in vitro*. Following a 3 to 24 hour-exposure to IFNβ, upregulated genes greatly outnumbered downregulated genes. All top 20 upregulated genes represent known IRGs previously identified in various cell types. The upregulated IRGs of PBMC were classified into several functional categories. The list included not only conventional IFN-response markers and components of IFN-signaling pathways, but also contained many proinflammatory chemokines and cytokines. This is surprising because IFNβ acts principally as an anti-Th1, anti-inflammatory cytokine [6,7]. By analyzing global gene expression profile, the present study for the first time showed that IFNβ induced a burst of gene expression of CXCR3 ligand chemokines (SCYB11, SCYB10 and SCYB9) and CCR2 ligand chemokines (SCYA8 and SCYA2), which was verified by quantitative real-time RT-PCR analysis. The chemokine genes actually have ISRE or IRE in the promoter regions, indicating direct targets of IFNβ[27,28].

CXCR3 is expressed predominantly on activated Th1 T cells, while CCR2 is expressed chiefly on monocytes [29]. The number of CXCR3^+ ^T cells is increased in the blood of RRMS, and they accumulate in perivascular infiltrates in active MS lesions [30,31], while SCYB10 (IP-10) and SCYB9 (MIG) are detected in the cerebrospinal fluid (CSF) of RRMS at acute relapse and expressed in reactive astrocytes in active MS lesions [31,32]. SCYA2 (MCP1) and SCYA8 (MCP2) immunoreactivities are also identified in reactive astrocytes in active demyelinating lesions of MS [33,34]. These observations suggest that CXCR3, CCR2, and their ligand chemokines positively regulate active inflammation in MS. Although the precise cell types expressing CXCR3 ligand and CCR2 ligand chemokines in PBMC in response to IFNβ remain to be characterized, the chemokine burst plays a central role in rapid activation and systemic recruitment of Th1 T cells and monocytes immediately after initiation of IFNβ treatment. A recent study showed that IFNβ promotes trafficking of mouse leukocytes by regulating a specific set of chemokines [35]. However, concurrent upregulation of a set of CXCR3 and CCR2 ligand chemokines has not previously been reported in MS patients on a long-term IFNβ treatment [16,17,20-22], suggesting that this phenomenon is an immediate early but transient event *in vivo*. IFNβ immediately reduced the expression of RGS14 (the most significantly downregulated gene at 3 hours; see Table 3), a member of the regulator of G protein signaling (RGS) gene family that acts as a negative regulator of G protein-coupled receptor (GPCR) signaling. Since all chemokine receptors are GPCR, IFNβ-induced downregulation of RGS14 might facilitate chemokine responsiveness in the cells expressing RGS14 [36,37]. Much less is known about the mechanism for regulation of IFNβ-repressed genes [38]. We identified IL-8 as one of IFNβ-repressed genes in PBMC (Table 3). IFNβ inhibits the transcription of IL-8 gene, possibly by binding of NF-κB repressing factor (NRF) to a negative regulatory element of the IL-8 promoter [39]. Serum IL-8 levels and IL-8 secretion from PBMC are elevated in untreated MS, and then reduced following IFNβ therapy [40]. Downregulation of IL-8 expression in PBMC during IFNβ treatment provides a predictive indicator for the responders in RRMS [20].

IFNβ also promptly upregulated a variety of proinflammatory cytokines, such as IL-6, IL-15, osteopontin, TNFα, and IFNγ in PBMC (Table 4). IFNβ promotes production of TNFα and IFNγ in unstimulated PBMC but decreases their levels in preactivated PBMC [41-43]. IFNβ increases the number of IFNγ-secreting cells *in vivo *at the early period of the treatment [44]. Most importantly, proinflammatory cytokines and chemokines induced by IFNβ have relevance to treatment-related early adverse effects. There exists a close relationship between flu-like symptoms and increased levels of IL-6 [45]. A single injection of IFNβ induces a transient burst of SCYB10 (IP-10) in the plasma of RRMS patients, which correlates with an incidence of flu-like symptoms [46]. IFNβ enhances the expression of CD80, SCYB10 (IP-10) and SCYA2 (MCP1) *in situ *at sites of injection, leading to chemotaxis of lymphocytes and monocytes in the lesions of skin reaction [47-49]. We found that IFNβ aberrantly regulated the levels of expression of several cytochrome P450 (CYP) enzymes (see Additional files 3,4,5,6). Type I IFN reduces the activity of CYP enzymes that metabolize various endogenous and exogenous substrates, probably leading to an increase in the potential for IFN-related hepatotoxicity [50].

Finally, the list of IRGs included various apoptosis regulators and HSP family members. ISRE-like sequences are identified in the regulatory element of CASP1, CASP4, CASP8, TNFRSF6 (FAS), TNFSF6 (FASL) and TNFSF10 (TRAIL), suggesting that IFNβ acts as a proapototic cytokine [51,52]. A recent study showed that early and sustained induction of TRAIL provides a marker for IFNβ treatment response in MS [53]. Furthermore, IFNβ-inducible apoptosis regulators play an immunoregulatory role. TNFR1-associated via death domain (TRADD) inhibits IFNγ-induced STAT1α activation [54]. Receptor-interacting serine-theronine kinase 1 (RIPK1) regulates TLR3-independent viral double-stranded RNA-induced type I IFN production [55]. Because HSPs in general act as an anti-apoptotic defender, the induction of HSP gene expression might occur as a counterbalance against upregulation of proapoptotic regulators. Alternatively, IRGs could directly enhance HSP expression. IFNβ-induced STAT1, by interacting with heat shock factor-1 (HSF1), activates the HSP70 and HSP90β gene promoters [56].

## Conclusion

Microarray analysis showed that IFNβ immediately induces a burst of gene expression of proinflammatory chemokines and cytokines *in vitro *that have potential relevance to IFNβ-related early adverse effects in MS patients *in vivo*.

## Abbreviations

MS = multiple sclerosis; IFNβ = interferon-beta; IRGs = IFNβ-responsive genes; PBMC = peripheral blood mononuclear cells; CNS = central nervous system; IFNγ = interferon-gamma; Th1 = T helper type 1; MHC = major histocompatibility complex; RRMS = relapsing-remitting multiple sclerosis; ISG = IFN stimulated gene; TLR = Toll-like receptor; STAT = signal transducer and activator of transcription; IRF = interferon regulatory factor; ISRE = interferon-stimulated response element; IRE = interferon regulatory factor-recognition element; HSPs = heat shock proteins; CSF = cerebrospinal fluid; GPCR = G protein-coupled receptor; RGS = regulator of G protein signaling.

## Competing interests

The author(s) declare that they have no competing interests.

## Authors' contributions

JS, YN and HT carried out DNA microarray and real-time RT-PCR analysis, and JS drafted the manuscript. TY participated in the design of the study and helped to draft the manuscript. All authors read and approved the final manuscript.

## Pre-publication history

The pre-publication history for this paper can be accessed here:



## Supplementary Material

Additional File 1 **The gene list of cDNA microarray utilized in the present study**. The complete gene list of cDNA microarray utilized in the present study is shown. It includes 1,258 well-annotated genes, selected from cytokines, growth factors and their receptors, apoptosis regulators, oncogenes, transcription factors, cell cycle regulators and housekeeping genes. Click here for file

Additional File 2 **Scatter plots of three distinct microarray experiments**. The figure represents a scatter plot exhibiting the comparison between the fluorescence intensity (FI) of Cy5 signals in the longitudinal axis and FI of Cy3 signals in the horizontal axis. (a) the subject #1 (a 46 year-old healthy man), (b) the subject #2 (a 28 year-old healthy man), and (c) the subject #4 (a 27 year-old woman with RRMS who was a dropout of IFNβ treatment due to induction of frequent severe relapses). Click here for file

Additional File 3 **The complete list of upregulated genes in PBMC following exposure to IFNβ for 3 hours**. Upregulated genes in PBMC of the subject #1 (a 46 year-old healthy man) by a 3 hour-exposure to 50 ng/ml recombinant human IFNβ are listed with Cy5/Cy3 signal intensity ratio, gene symbol, GenBank accession number, and gene name. In vivo IRG in T cells and non-T cells of RRMS patients reported previously (Ref. 16) are underlined. Click here for file

Additional File 4 **The complete list of downregulated genes in PBMC following exposure to IFNβ for 3 hours**. Downregulated genes in PBMC of the subject #1 (a 46 year-old healthy man) by a 3 hour-exposure to 50 ng/ml recombinant human IFNβ are listed with Cy5/Cy3 signal intensity ratio, gene symbol, GenBank accession number, and gene name. Click here for file

Additional File 5 **The complete list of upregulated genes in PBMC following exposure to IFNβ for 24 hours**. Upregulated genes in PBMC of the subject #1 (a 46 year-old healthy man) by a 24 hour-exposure to 50 ng/ml recombinant human IFNβ are listed with Cy5/Cy3 signal intensity ratio, gene symbol, GenBank accession number, and gene name. In vivo IRG in T cells and non-T cells of RRMS patients reported previously (Ref. 16) are underlined. Click here for file

Additional File 6 **The complete list of downregulated genes in PBMC following exposure to IFNβ for 24 hours**. Downregulated genes in PBMC of the subject #1 (a 46 year-old healthy man) by a 24 hour-exposure to 50 ng/ml recombinant human IFNβ are listed with Cy5/Cy3 signal intensity ratio, gene symbol, GenBank accession number, and gene name. Click here for file

Additional File 7 **Top 20 upregulated genes in PBMC following exposure to IFNβ for 3 hours: two additional subjects**. Upregulated genes in PBMC of the subject #2 (a 28 year-old healthy man) and #4 (a 27 year-old woman with RRMS who was a dropout of IFNβ treatment due to induction of frequent severe relapses) following a 3 hour-exposure to 50 ng/ml recombinant human IFNβ are listed with Cy5/Cy3 signal intensity ratio, gene symbol, and gene name. Both CXCR3 ligand (yellow) and CCR2 ligand (blue) chemokines are highlighted. Click here for file

## Figures and Tables

**Table 1:** Primers utilized for real-time RT-PCR analysis

Genes	GenBank accession No.	Sense primers	Antisense primers	PCR product (bp)
ISG15 (G1P2)	NM_005101 (M13755)	5'aagcccctgagcaccgtgttcat3'	5'ttgatcctgctcggatgctggtg3'	102
SCYB10 (CXCL10, IP-10)	NM_001565 (X02530)	5'tcgatgcagtgcttccaaggatgg3'	5'ccttcctacaggagtagtagcagc3'	162
SCYA8 (CCL8, MCP2)	NM_005623 (Y10802)	5'tctgtgctgaccccaaggagagat3'	5'taatgtcacactgcacctggggga3'	164
SCYA2 (CCL2, MCP1)	NM_002982 (S71513)	5'ctagctttccccagacaccctgtt3'	5'ccaggggtagaactgtggttcaag3'	197
SCYB2 (CXCL2, GRO2)	NM_002089	5'cccgcatcgcccatggttaagaaa3'	5'tcttctgttcctgtaaggcagggc3'	131
FOS	NM_005252	5'gagctggtgcattacagagaggag3'	5'ggacttgagtccacacatggatgc3'	140
RGS14	NM_006480	5'tgacagctacccaacagtccagga3'	5'agggattgggggtgagcttgttga3'	222
G3PDH	NM_002046	5'ccatgttcgtcatgggtgtgaacca3'	5'gccagtagaggcagggatgatgttc3'	251

Abbreviations: ISG15, interferon-stimulated gene 15; SCYB10, small inducible cytokine subfamily B, member 10; SCYA8, small inducible cytokine subfamily A, member 8; SCYA2, small inducible cytokine subfamily A, member 2; SCYB2, small inducible cytokine subfamily B, member 2; FOS, cellular oncogene c-fos; RGS14, regulator of G-protein signaling 14; and G3PDH, glyceraldehyde-3-phosphate dehydrogenase

**Table 2:** Top 20 upregulated genes in PBMC following exposue to IFNβ

No.	Cy5/Cy3 ratio	Symbol	GenBank	Gene name
at 3 hours				
1	188.50	IFIT1	X03557	interferon-induced protein with tetratricopeptide repeats 1 (IFI56)
2	149.82	SCYB11	AF030514	chemokine (C-X-C motif) ligand 11 (CXCL11, IP-9, I-TAC)
3	81.81	ISG15	M13755	interferon-stimulated gene ISG15 (G1P2)
4	67.84	IFIT4	AF083470	interferon-induced protein with tetratricopeptide repeats 4 (IFI60)
5	66.38	MX1	M30817	myxovirus resistance protein 1 (MXA)
6	64.43	SCYB10	X02530	chemokine (C-X-C motif) ligand 10 (CXCL10, IP-10)
7	55.01	SCYA8	Y10802	chemokine (C-C motif) ligand 8 (CCL8, MCP2)
8	36.88	SCYB9	X72755	chemokine (C-X-C motif) ligand 9 (CXCL9, MIG)
9	35.14	TNFSF10	U37518	tumor necrosis factor superfamily, member 10 (TRAIL)
10	32.86	MX2	M30818	myxovirus resistance protein 2 (MXB)
11	21.99	IFI27	X67325	interferon alpha-inducible protein 27
12	21.98	G1P3	X02492	interferon alpha-inducible protein (IFI6-16), isoform a
13	19.26	ISG20	U88964	interferon-stimulated gene ISG20
14	19.12	STAT1	M97935	signal transducer and activator of transcription 1, isoform alpha
15	13.71	IRF7	U53830	interferon-regulatory factor 7, isoform alpha
16	13.44	CCNA1	U66838	cyclin A1
17	12.72	IL6	M14584	interleukin 6
18	10.86	IL1RN	X53296	interleukin 1 receptor antagonist, isoform 1
19	10.47	SCYA2	S71513	chemokine (C-C motif) ligand 2 (CCL2, MCP1)
20	9.91	STAT2	M97934	signal transducer and activator of transcription 2
at 24 hours				
1	193.19	SCYA8	Y10802	chemokine (C-C motif) ligand 8 (CCL8, MCP2)
2	124.77	IFIT1	X03557	interferon-induced protein with tetratricopeptide repeats 1 (IFI56)
3	107.21	IFI27	X67325	interferon alpha-inducible protein 27
4	70.51	ISG15	M13755	interferon-stimulated gene ISG15 (G1P2)
5	53.22	SCYB11	AF030514	chemokine (C-X-C motif) ligand 11 (CXCL11, IP-9, I-TAC)
6	38.07	MX1	M30817	myxovirus resistance protein 1 (MXA)
7	37.95	IFIT4	AF083470	interferon-induced protein with tetratricopeptide repeats 4 (IFI60)
8	31.56	SCYB10	X02530	chemokine (C-X-C motif) ligand 10 (CXCL10, IP-10)
9	23.65	G1P3	X02492	interferon alpha-inducible protein (IFI6-16), isoform a
10	23.10	TNFSF10	U37518	tumor necrosis factor superfamily, member 10 (TRAIL)
11	21.31	MX2	M30818	myxovirus resistance protein 2 (MXB)
12	20.68	PDGFRL	D37965	platelet-derived growth factor receptor-like protein
13	15.95	CD80	M27533	CD80 antigen (B7-1)
14	13.99	IRF7	U53830	interferon-regulatory factor 7, isoform alpha
15	13.93	IL1RN	X53296	interleukin 1 receptor antagonist, isoform 1
16	13.31	ISG20	U88964	interferon-stimulated gene ISG20
17	11.81	IFITM1	J04164	interferon induced transmembrane protein 1 (IFI17)
18	8.67	SCYA2	S71513	chemokine (C-C motif) ligand 2 (CCL2, MCP1)
19	8.54	IL6	M14584	interleukin 6
20	8.51	IL8RB	L19593	interleukin 8 receptor beta (CXCR2)

Top 20 upregulated genes in PBMC of the subject #1 (a 46 year-old healthy man) by exposure to 50 ng/ml recombinant human IFNβ for 3 or 24 hours are listed with Cy5/Cy3 signal intensity ratio, gene symbol, GenBank accession number, and gene name. In vivo IRGs in T cells and non-T cells of RRMS patients reported previously (Ref. 16) are underlined.

**Table 3:** Top 20 downregulated genes in PBMC following exposue to IFNβ

No.	Cy5/Cy3 ratio	Symbol	GenBank	Gene name
at 3 hours				
1	0.29	RGS14	NM_006480	regulator of G-protein signaling 14
2	0.30	FOS	NM_005252	cellular oncogene c-fos
3	0.31	SCYB2	NM_002089	chemokine (C-X-C motif) ligand 2 (GRO2, MIP2A)
4	0.41	PPARG2	U63415	peroxisome proliferative activated receptor gamma, isoform 2
5	0.41	TNFSF11	NM_003701	tumor necrosis factor ligand superfamily, member 11 (RANKL), isoform 1
6	0.42	ABCC4	NM_005845	ATP-binding cassette, subfamily C, member 4 (MRP4)
7	0.42	MERTK	NM_006343	c-mer proto-oncogene tyrosine kinase
8	0.42	TGFBR3	NM_003243	transforming growth factor beta receptor III (betaglycan)
9	0.44	ATP2B1	J04027	ATPase, Ca++ transporting, plasma membrane 1, isoform 1b
10	0.45	SCYB1	NM_001511	chemokine (C-X-C motif) ligand 1 (GRO1, MGSA)
11	0.45	IL8	NM_000584	interleukin 8 (SCYB8, CXCL8)
12	0.45	TRAF5	NM_004619	TNF receptor-associated factor 5, variant 1
13	0.47	CD3G	NM_000073	CD3G antigen, gamma polypeptide
14	0.47	IL1A	NM_000575	interleukin 1 alpha
15	0.47	TCFL5	NM_006602	transcription factor-like 5
16	0.48	LAT	AF036905	linker for activation of T cells, isoform b
17	0.48	HNMT	U08092	histamine N-methyltransferase, isoform 1
18	0.48	SLC3A1	NM_000341	solute carrier family 3, member 1
19	0.49	MGST1	NM_145764	microsomal glutathione S-transferase 1, variant 1d
20	0.49	TNFSF8	NM_001244	tumor necrosis factor ligand superfamily, member 8 (CD30L)
at 24 hours				
1	0.18	ABCC3	AF083552	ATP-binding cassette, subfamily C, member 3, isoform MRP3
2	0.20	FOS	NM_005252	cellular oncogene c-fos
3	0.30	ALDH2	NM_000690	aldehyde dehydrogenase 2 family
4	0.30	TPST1	NM_003596	tyrosylprotein sulfotransferase 1
5	0.31	CD9	NM_001769	CD9 antigen
6	0.34	NRG1	NM_013962	neuregulin 1, isoform GGF2
7	0.35	TLR5	NM_003268	toll-like receptor 5
8	0.36	PTGS1	NM_000962	prostaglandin-endoperoxide synthase 1 (COX1), isoform 1
9	0.37	MGST2	NM_002413	microsomal glutathione S-transferase 2
10	0.38	ITGAM	NM_000632	integrin, alpha M (CD11B, MAC-1, CR3A)
11	0.38	SCYA24	NM_002991	chemokine (C-C motif) ligand 24 (eotaxin 2)
12	0.38	CYP27A1	NM_000784	cytochrome P450, family 27, subfamily A, polypeptide 1 (CTX)
13	0.39	ALDH1A1	NM_000689	aldehyde dehydrogenase 1 family, member A1
14	0.39	MYCL1	NM_005376	v-myc myelocytomatosis viral oncogene homolog 1 (LMYC)
15	0.41	ATP6AP2	NM_005765	ATPase, H+ transporting, lysosomal accessory protein 2 (ATP6M8-9)
16	0.44	PRKCG	NM_002739	protein kinase C gamma
17	0.45	ATP2B1	J04027	ATPase, Ca++ transporting, plasma membrane 1, isoform 1b
18	0.46	AP1S2	NM_003916	adaptor-related protein complex 1, sigma 2 subunit
19	0.47	NR6A1	NM_001489	nuclear receptor subfamily 6, group A, member 1 (RTR), isoform 2
20	0.47	GCG	NM_173916	glucagon

Top 20 downregulated genes in PBMC of the subject #1 (a 46 year-old healthy man) by exposure to 50 ng/ml recombinant human IFNβ for 3 or 24 hours are listed with Cy5/Cy3 signal intensity ratio, gene symbol, GenBank accession number, and gene name.

**Table 4:** Functional classification of IFNβ-upregulated genes in PBMC

**Categories**		**Number of genes**	**Gene symbols (alternative symbols or names)**
**1**	**Conventional IFN-response markers**	12	IFIT1 (IFI56), ISG15 (G1P2), IFIT4 (IFI60), MX1 (MXA), MX2 (MXB), IFI27, G1P3 (IFI6-16), ISG20, IFI16, IFITM1 (IFI17), IFITM3 (1-8U), ABCB2 (TAP1)
**2**	**Components of IFN-signaling pathways**	12	STAT1, IRF7, STAT2, JAK2, IRF2, ISGF3G (IRF9), MYD88, IRF8, STAT3, JAK3, IRF1, TLR3
**3**	**Chemokines and receptors**	11	SCYB11 (CXCL11, I-TAC), SCYB10 (CXCL10, IP-10), SCYA8 (CCL8, MCP2), SCYB9 (CXCL9, MIG), SCYA2 (CCL2, MCP1), CCR5, SCYA4 (CCL4, MIP1B), IL8RB (CXCR2), SCYA3 (CCL3, MIP1A), SCYA19 (CCL19, MIP3B), SCYA13 (CCL13, MCP4)
**4**	**Cytokines, growth factors, and receptors**	17	IL6, ILRN (IL-1 receptor antagonist), IL1R2, IL15RA, IL15, SPP1 (osteopontin), CSF1, IL12RB2, TNF (TNFA), IL2RB, IFNG, NTRK1 (TRKA), PDGFRL, TNFAIP6, KITLG (SCF), IL10, IL3RA
**5**	**Apotosis, DNA damage, and cell cycle regulators**	29	TNFSF10 (TRAIL), CASP10, BAG1, TNFRSF6 (FAS), CASP4, TRADD, GZMA, CASP7, RIPK2, MAD, RIPK1, CFLAR (FLIP), RELA, STK3, CASP1, TNFSF6 (FASL), PARP4, TANK (I-TRAF), POLE2, LMNB1, E2F2, CCNA1 (cyclin A1), CDKN1A (p21), PPP1R15A (GADD34), CASP3, CDKN1C (p57), CDK5R2 (p39), TERF1, NBS1 (nibrin)
**6**	**Heat shock proteins**	9	HSPA6 (HSP70B'), HSJ2 (HSPF4), HSPA1A (HSP70-1), HSPA1B (HSP70-2), HSPCA (HSP90A), HSPA5 (GRP78), HSPA1L (HSP70-HOM), HSPA8 (HSC70), HSPB1 (HSP27)
**7**	**Costimulatory and adhesion molecules**	7	CD80 (B7-1), SELL (selectin L), TNFRSF5 (CD40), CD163, CD86 (B7-2), HLA-DRA, FCER1G

The upregulated IRGs in PBMC listed in Additional files 3 and 5 were classified into seven functional categories.
