# Supplementary figures and images for: Microarray analysis identifies a set of CXCR3 and CCR2 ligand chemokines as early IFNβ-responsive genes in peripheral blood lymphocytes in vitro: an implication for IFNβ-related adverse effects in multiple sclerosis
Source: BMC Neurol. 2006 May 19;6:18. doi: 10.1186/1471-2377-6-18 (PMC1483835; doi:10.1186/1471-2377-6-18)

## Slide 1
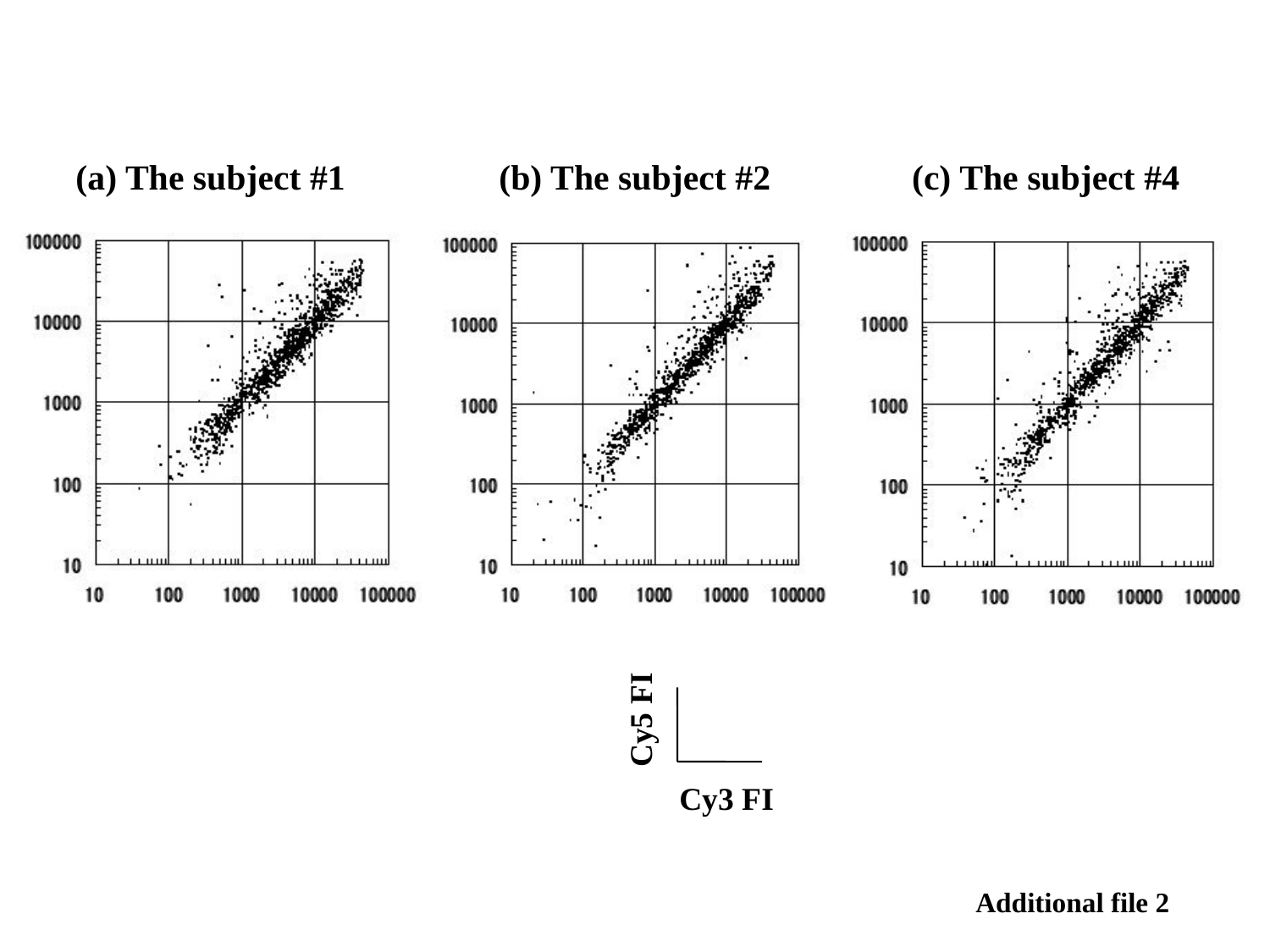

(a) The subject #1
(b) The subject #2
(c) The subject #4
Cy5 FI
Cy3 FI
Additional file 2

Supplement: Additional File 2 — Scatter plots of three distinct microarray experiments. The figure represents a scatter plot exhibiting the comparison between the fluorescence intensity (FI) of Cy5 signals in the longitudinal axis and FI of Cy3 signals in the horizontal axis. (a) the subject #1 (a 46 year-old healthy man), (b) the subject #2 (a 28 year-old healthy man), and (c) the subject #4 (a 27 year-old woman with RRMS who was a dropout of IFNβ treatment due to induction of frequent severe relapses). [file 1471-2377-6-18-S2.ppt]
